# Massive spontaneous pneumothorax with mediastinal shift in a 14-year-old with pulmonary tuberculosis: A case report

**DOI:** 10.1016/j.ijscr.2025.111422

**Published:** 2025-05-09

**Authors:** Abdisalam Ahmed Sandeyl, Mohamed Jayte, Mumbere Mayani David, Abishir Mohamud Hirsi, Zakaria Abdullahi Hussein, Abdisamad Guled Hersi

**Affiliations:** Internal Medicine Department at Kampala International University, Kampala, Uganda

**Keywords:** Spontaneous pneumothorax, Pulmonary tuberculosis, Secondary pneumothorax, *Mycobacterium tuberculosis*, Chronic cough

## Abstract

**Introduction and importance:**

Spontaneous pneumothorax is a life-threatening complication of pulmonary tuberculosis (TB), particularly in endemic regions. Secondary spontaneous pneumothorax (SSP) occurs due to underlying lung pathology, such as cavitary lesions in TB. This case highlights the presentation, diagnosis, and management of SSP in a TB-endemic setting.

**Case presentation:**

A 14-year-old male, presented with acute dyspnea, pleuritic chest pain, and a history of chronic cough and weight loss. Clinical examination revealed diminished breath sounds and hyperresonance on the left hemithorax. Imaging confirmed a massive left-sided pneumothorax with cavitary lesions. Sputum analysis confirmed *Mycobacterium tuberculosis*. Immediate chest tube insertion and antitubercular therapy (ATT) were initiated, resulting in clinical improvement.

**Clinical discussion:**

SSP in TB patients is a rare but serious complication, often resulting from rupture of subpleural tuberculous cavities into the pleural space. Delayed diagnosis can lead to tension pneumothorax, respiratory failure, and increased mortality. Imaging, particularly chest X-ray and computed tomography (CT), plays a crucial role in diagnosis. Management includes prompt chest tube insertion for lung re-expansion and ATT to address the underlying TB infection. In resource-limited settings, where advanced surgical interventions such as pleurodesis and video-assisted thoracoscopic surgery (VATS) may not be readily available, early medical intervention remains the cornerstone of treatment.

**Conclusion:**

This case underscores the importance of early recognition and management of SSP in TB patients, particularly in resource-limited settings. Prompt chest tube insertion and ATT are critical to preventing morbidity and mortality.

## Highlights

• Secondary spontaneous pneumothorax (SSP) is a severe complication of pulmonary TB.
• Early recognition and management of SSP in TB-endemic settings are critical.
• Chest tube insertion and antitubercular therapy (ATT) are essential for treatment.
• Imaging plays a crucial role in diagnosing SSP in resource-limited settings.
• Timely intervention prevents morbidity and mortality in affected patients.

## 1 Introduction

Spontaneous pneumothorax is the accumulation of air in the pleural space without preceding trauma or iatrogenic cause. It is classified into primary spontaneous pneumothorax (PSP), which occurs in individuals without clinically apparent lung disease, and secondary spontaneous pneumothorax (SSP), which occurs in the context of underlying pulmonary pathology such as chronic obstructive pulmonary disease (COPD), cystic fibrosis, or pulmonary tuberculosis (TB) [1].

Pulmonary TB is a leading cause of SSP, particularly in endemic regions. The disease causes parenchymal destruction, cavitation, and subpleural bleb formation, which predispose to air leakage into the pleural space [2]. Although SSP is a well-documented complication of TB, it is relatively rare, occurring in approximately 1–2 % of TB cases [3]. However, its clinical importance cannot be overstated, as it often indicates advanced disease and carries a higher risk of morbidity and mortality, particularly in resource-limited settings where diagnostic and therapeutic interventions are often delayed [4].

Globally, TB remains a significant public health challenge, with an estimated 10 million cases and 1.5 million deaths in 2022 [5]. Sub-Saharan Africa bears the highest burden, accounting for 23 % of global cases, with Uganda ranking among the high-burden countries [6]. In Uganda, TB prevalence is exacerbated by limited healthcare resources, delayed diagnosis, and high rates of HIV co-infection [7].

This case report aims to highlight the clinical presentation, diagnostic challenges, and management of SSP complicating pulmonary TB in a 14-year-old. The objective is to emphasize the importance of early recognition and prompt intervention in resource-limited settings.

## 2 Case presentation

A 14-year-old male come with a 2-day history of acute-onset dyspnea and left-sided pleuritic chest pain. He reported a 3-week history of productive cough, fever, night sweats, and significant weight loss (approximately 5 kg over the past month). There was no history of trauma, smoking, or prior lung disease. The patient lived in a rural, TB-endemic area with limited access to healthcare. He had no known contact with TB patients and no family history of TB or other chronic lung diseases.

On admission, the patient appeared acutely ill, exhibiting visible respiratory distress. His respiratory rate was 28 breaths per minute, heart rate 110 beats per minute, blood pressure 100/60 mmHg, temperature 38.2 °C, and oxygen saturation 88 % on room air.

**Fig. 1:**
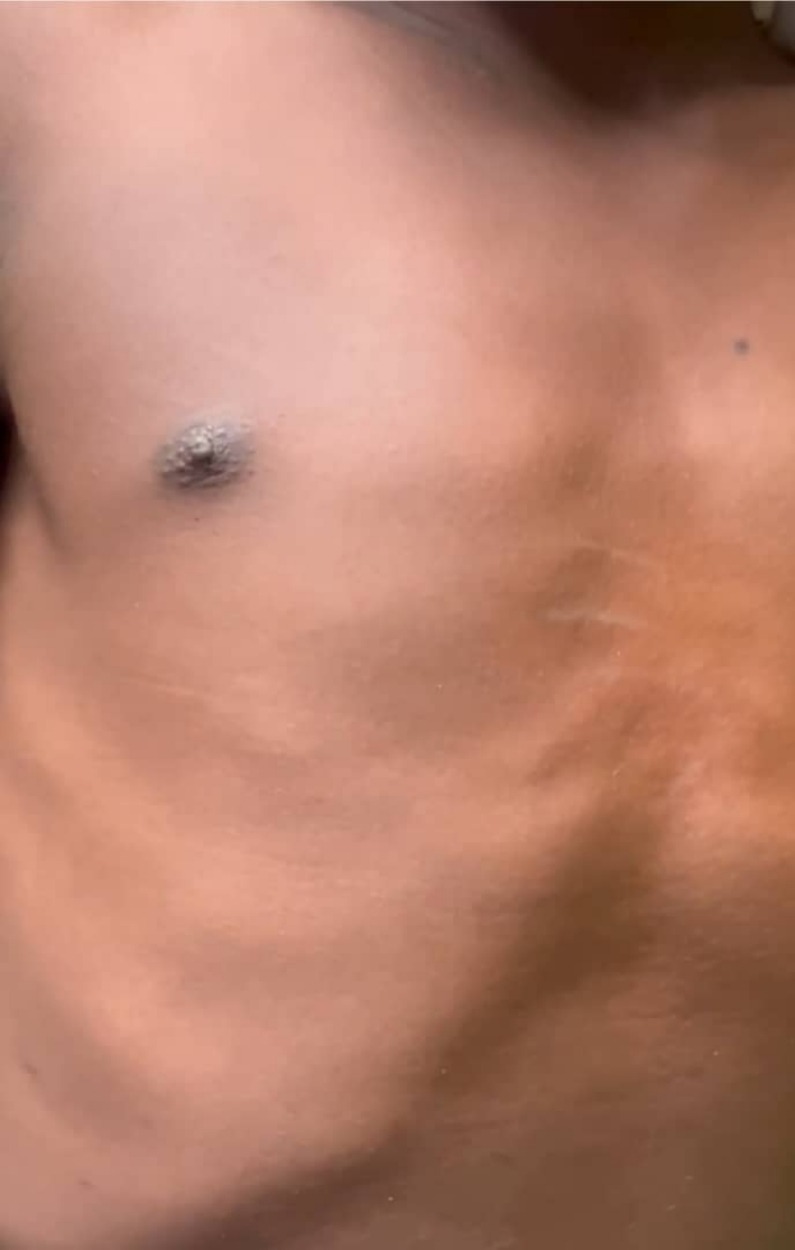
Showing diminished breath sounds over the left hemithorax, hyperresonance on percussion, and no audible wheezing or crackles.

Physical examination revealed diminished breath sounds over the left hemithorax, hyperresonance on percussion, and no audible wheezing or crackles (Fig. 1). Cardiovascular assessment showed tachycardia with normal heart sounds and no murmurs. There was no lymphadenopathy, hepatosplenomegaly, or signs of extrapulmonary tuberculosis.

**Fig. 2:**
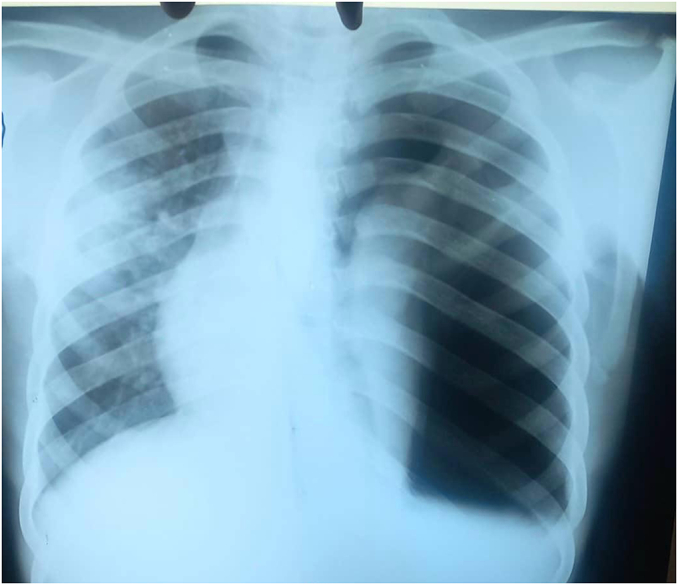
Showing A chest X-ray of a massive left-sided pneumothorax with 60 % lung collapse, cavitary lesions in the upper lobe, and a rightward mediastinal shift mimicking dextrocardia.

Given the clinical presentation, a provisional diagnosis of spontaneous pneumothorax secondary to pulmonary tuberculosis was made. A chest X-ray showed a massive left-sided pneumothorax with 60 % lung collapse, cavitary lesions in the upper lobe, and a rightward mediastinal shift mimicking dextrocardia (Fig. 1). Sputum analysis identified acid-fast bacilli on Ziehl-Neelsen staining, and nucleic acid amplification testing confirmed *M. tuberculosis*. Laboratory tests (Table 1) revealed an elevated erythrocyte sedimentation rate of 65 mm/h (normal: <20 mm/h) and an elevated C-reactive protein level of 45 mg/L (normal: <10 mg/L). The HIV test was negative. Due to the unavailability of CT scan facilities at Kisoro General Hospital, further imaging was not performed (Fig. 2).

**Table 1:** Showing comparing laboratory test results from day 1 up to day 7.

Test	Admission	Day 7	Normal range
ESR (mm/h)	65	40	<20
CRP (mg/L)	45	20	<10
Hemoglobin (g/dL)	10.5	11.2	12–16
White cell count (×10^3^/μL)	12.0	8.5	4–11

**Fig. 3:**
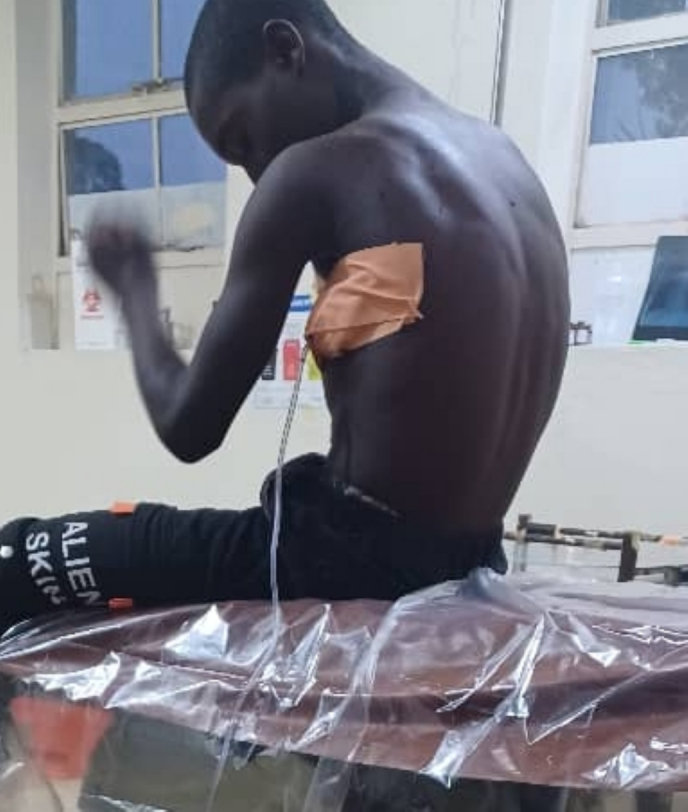
Showing A 28-French chest tube inserted to the patient as chest tube.

A 28-French chest tube (Fig. 3) was inserted under aseptic conditions, as larger-bore tubes are preferred in massive secondary pneumothorax with suspected cavitary TB, to facilitate better drainage and reduce risk of tube blockage. Although smaller-bore chest tubes have gained popularity for primary pneumothorax, the British Thoracic Society guidelines recommend larger sizes (≥24 Fr) in cases with ongoing air leak, hemothorax, or infection risk. Antitubercular therapy was initiated with a standard four-drug regimen consisting of isoniazid, rifampin, pyrazinamide, and ethambutol following confirmation of tuberculosis. Supportive care included oxygen therapy, analgesics, and nutritional support.

## 3 Outcome and follow-up

The patient showed significant clinical improvement (Table 2) over the next seven days. Follow-up lab test demonstrated resolution of the pneumothorax and partial healing of cavitary lesions. He was discharged with a plan for six months of antitubercular therapy and scheduled for regular follow-up. However, he lost follow-up, and his treatment outcome remains unknown.

**Table 2:** Showing clinical improvement of the patient from admission to discharge.

Parameter	Admission	Discharge
Respiratory rate (/min)	28	18
Oxygen saturation (%)	88	96
Chest pain	Severe	Mild
Cough	Severe	Moderate
Lung expansion	60 % collapse	Fully expanded

This case report has been prepared in accordance with the Surgical CAse REport (SCARE) 2023 guidelines [11].

## 4 Discussion

This case illustrates the classic presentation of SSP complicating pulmonary TB in a young male from a TB-endemic region. The patient's symptoms, imaging findings, and sputum analysis were consistent with advanced TB and pneumothorax. Prompt chest tube insertion and ATT were lifesaving.

Pulmonary TB leads to SSP through several mechanisms, including rupture of subpleural caseous nodules, cavitary lesions, or bronchopleural fistulas. The inflammatory process weakens the lung parenchyma, increasing the risk of air leakage into the pleural space [8].

Diagnosing SSP in TB patients can be challenging due to overlapping symptoms such as cough and dyspnea. Imaging plays a crucial role in differentiating pneumothorax from other complications like pleural effusion or empyema [9]. In this case, the unavailability of CT scan facilities highlights the diagnostic challenges faced in resource-limited settings. In similar low-resource settings, where advanced surgical interventions such as VATS or pleurodesis are unavailable, alternative options such as prolonged tube thoracostomy with a Heimlich valve have been reported to be effective. These approaches provide continued one-way drainage of pleural air and can reduce hospital stay while awaiting definitive recovery or referral.

Similar cases have been reported in TB-endemic regions. A study from India found that 15 % of SSP cases were attributable to TB, with young males being disproportionately affected [10]. Another case series from South Africa highlighted the high morbidity and mortality associated with TB-related pneumothorax in HIV-coinfected patients [10].

## 5 Conclusion

This case underscores the importance of early recognition and management of SSP complicating pulmonary TB, particularly in resource-limited settings. Key lessons include the need for heightened clinical suspicion in TB-endemic regions, prompt chest tube insertion, and timely initiation of ATT. Strengthening TB diagnosis and treatment programs in endemic regions is essential to reducing complications like pneumothorax and improving patient outcomes.

## Author contribution

Study conception and design: Abdisalam Ahmed Sandeyl, Mohamed Jayte, Mumbere Mayani David.

Data collection and case management: Abishir Mohamud Hirsi, Zakaria Abdullahi Hussein.

Manuscript drafting and literature review: Abdisamad Guled Hersi.

Critical revision and final approval: All authors.

## Consent for publication

Written informed consent was obtained from the patient's parents/legal guardian for publication and any accompanying images. A copy of the written consent is available for review by the Editor-in-Chief of this journal on request.

## Ethical approval

Ethical approval to report this case was obtained from the Ethics Committee at Kisoro General Hospital (Approval Number: KH-EC-2024-12-02).

## Guarantor

Mohamed Jayte.

Abdisalam Ahmed Sandeyl.

## Research registration number

Not applicable.

## Funding

This research received no specific grant from any funding agency, commercial, or not-for-profit sectors.

## Conflict of interest statement

The authors declare no conflicts of interest associated with this study.

## Data Availability

Not applicable.
